# Case Report of *Hemomania*: Recurrent Bloodletting and Blood Consumption From Self and Others

**DOI:** 10.1155/crps/6417116

**Published:** 2026-07-22

**Authors:** Sifat E. Syed, Nishat Tamanna Nur, Shabnam Saba, Nahid Mahjabin Morshed

**Affiliations:** ^1^ Department of Psychiatry, Bangladesh Medical University, Dhaka, Bangladesh

**Keywords:** Bangladesh, bloodletting, impulse control disorders, self-injurious behavior

## Abstract

*Hemomania* is a proposed term for incontrollable urges to see, release, or ingest blood which is not a formally recognized diagnostic entity. This behavioral phenomenon overlaps with nonsuicidal self‐injury (NSSI), impulse control disorders, and elements of clinical vampirism. We present the case of a 22‐year‐old female with a longstanding history of bloodletting using syringes, blood ingestion, and collection. The patient collected blood in vials, sometimes mixed them with water and drank it. She also collected and consumed blood from other individuals and animals occasionally. These behaviors did not align with existing diagnostic categories, therefore, conceptualized within the proposed construct of *hemomania*. She had comorbid major depressive disorder with psychotic features, borderline personality disorder (BPD), and schizotypal traits. Her problems intensified following recent marital discord, which acted as a key emotional trigger. Treatment comprising pharmacotherapy and psychotherapy resulted in partial improvement of symptoms but relapsed within a few months. This case describes an atypical, chronic presentations of exceptionally rare pattern of behavior and highlights the conventional diagnostic and therapeutic challenges underscoring the need for precise diagnostic framing, thorough differential assessment, and individualized treatment approaches for such complex psychiatric presentations.

## 1. Introduction

The desire to see blood is frequently detected among individuals engaging in nonsuicidal self‐injury (NSSI), who describe it as a source of comfort or relief [1]. However, risky behaviors such as deliberate bloodletting (withdrawal of blood using a syringe) and ingestion of blood are rare and clinically concerning. Recently, such behaviors have been theoretically grouped under the proposed term “*hemomania*,” which includes the incontrollable urge to see, release, or consume one’s own blood [2]. The term “*hemomania*” was first used and proposed by Kandeğer et al. [2] in their paper to describe two cases who had urges of seeing their own blood, bloodletting, and tasting/drinking blood. However, *hemomania* is not a formally recognized diagnostic entity in DSM‐5 or ICD classifications and remains a theoretical and poorly operationalized concept with limited empirical validation.

Symptomatology of *hemomania* such as compulsive bloodletting partially overlaps with features of NSSI which is a transdiagnostic behavioral phenomenon observed across several psychiatric disorders. However, “nonsuicidal self‐injury disorder” (NSSID) is a proposed diagnostic entity in DSM 5 and included as a condition for further study, rather than a formal diagnosis [3], underscoring the evolving understanding of self‐injurious behaviors [4]. Some authors have proposed that these behaviors may fall within the spectrum of impulse control disorders due to observed associations with impulsivity and impaired behavioral regulation [2]. *Hemomania* has been reported in individuals with various psychiatric diagnoses, including major depressive disorder, borderline personality disorder (BPD), antisocial personality disorder, anxiety disorders, dissociative disorders, adjustment disorder, and obsessive–compulsive and related disorders [5]. This heterogeneity further underscores the lack of diagnostic specificity and the current conceptual ambiguity surrounding *hemomania*. Persistent bloodletting and blood‐drinking behaviors present notable clinical risks as persistent bloodletting can result in factitious anemia [6] and even death in severe cases. While blood‐drinking behaviors are often referred to as folklore and mythology, several documented clinical cases underscore the need for further exploration and clinical awareness of this rare but dangerous phenomenon.

The aim of this case report is to describe a highly rare pattern of bloodletting, collection, and blood drinking from self and other individuals that does not fit existing diagnostic categories hence conceptualized within the proposed construct of *Hemomania*. It further highlights the complex interplay of mood, personality, and impulse control and underscores the diagnostic and therapeutic challenges clinicians may face in managing such atypical and chronic presentations.

This case report is based on a detailed clinical assessment during the patient’s inpatient admission. Information was collected through several separate interviews with the patient, as well as from her husband and family members, along with a review of the medical records. The psychiatric team was directly involved in day‐to‐day follow‐up including diagnosis, treatment, and further progress.

## 2. Case Presentation

Ms. A, a 22‐year‐old married woman, was admitted to the Bangladesh Medical University on 2024 with multiple somatic complaints of headache, abdominal pain, generalized aches, reduced appetite, and significant weight loss (17 kg). Physical examination and investigations revealed a very low body mass index (13.7 kg/m^2^), a hemoglobin level of 11.6 g/dL, and hematocrit of 35.1%. The peripheral blood film revealed microcytic hypochromic anemia, and she had hemoglobin E trait. After excluding major medical conditions like tuberculosis, she was referred and transferred to psychiatry.

**Figure 1:**
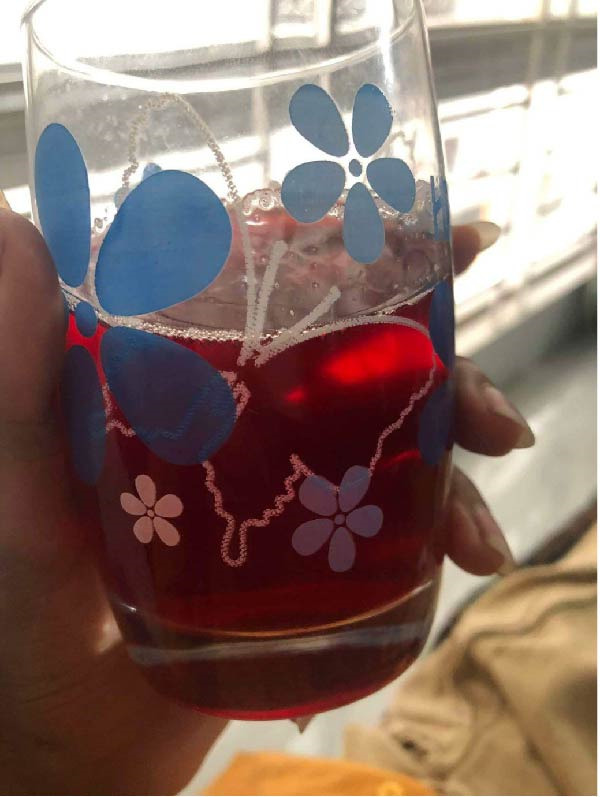
Mixing blood with water before drinking to get a larger volume.

**Figure 2:**
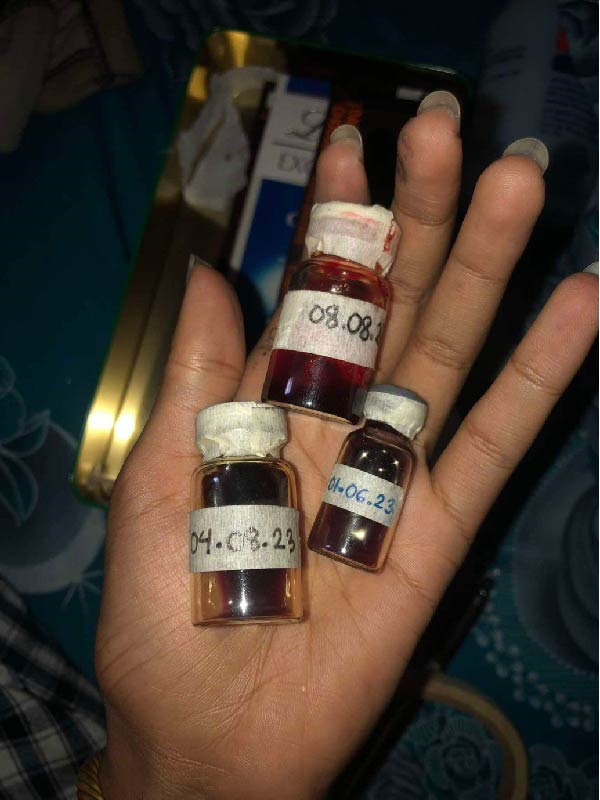
Collection of blood in vials with dates of collection.

Psychiatric evaluation revealed a history of frequent bloodletting and blood consumption. This began at the age of 10, when she unintentionally tasted her own blood and experienced stress relief. By the age of 12, she learned to extract blood using syringes and ingested frequently, especially when under stress. Sometimes she diluted blood with water and drank it to create the illusion of a larger volume (Figure 1). The act brought gratification and calmness, and she did not perceive the urges as distressing. She often recorded videos, painted with blood, and labeled blood vials for later use (Figure 2). She had a strong fascination with the smell, color, and taste of blood and occasionally linked ingestion to sexual arousal. She infrequently drew blood from her sister and boyfriend. She reported two episodes involving harm to pet animals during which she consumed their blood, for which she later expressed remorse. Despite her moral awareness, she described the urges as uncontrollable.

There were adverse childhood experiences like sibling rivalry and sexual and physical abuse. A history of a possible functional neurological symptom episode and psychiatric consultation at the age of 5 was reported by her father, though there were no supporting medical records. This episode may have been a functional neurological episode (as there was no features of true seizure), though unconfirmed. She has a history of emotional instability, self‐harm, and depressive symptoms since early adolescence. An interview with her family displayed magical beliefs; her father and sisters claimed to have telepathic powers. Her father claimed to foresee the future and the ability to access other galaxies through meditation and was a spiritual guide to his daughters. Upon knowing about the bloodletting behavior of their daughter, the family normalized it, focusing instead on her physical health. However, there is no evidence to conclude that the behaviors were cult or family‐mediated.

**Figure 3:**
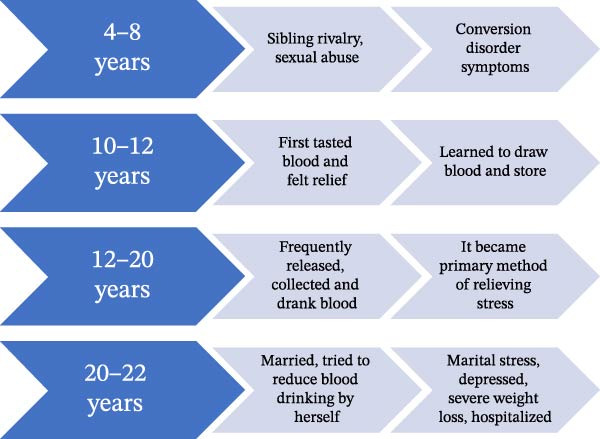
Timeline of key life events and clinical presentations.

At the age of 15, following her boyfriend’s suicide, she made several suicide attempts (hanging, poisoning, and wrist‐cutting), which coincided with worsening depression and increased bloodletting and blood drinking behavior. She consulted a number of psychiatrists but had poor treatment adherence. There was a brief history of substance use (cannabis, nicotine, and alcohol), which she later discontinued. She used her blood in secret artworks and maintained an academic performance, completing higher secondary education and excelling in creative pursuits. She married her boyfriend 2 years back and described her married life as complicated. Though she is aware of the health risks, she could not stop her blood‐related behaviors but reduced urges of blood drinking by rewatching photos/videos of bloodletting. She recently had serious marital discord, which led to deterioration of depression and intensified urges of bloodletting (Figure 3).

A mental state examination revealed that her mood was severely depressed with a congruent affect, and speech was slow and monotonous. She had negative automatic thoughts, persecutory delusions, and auditory hallucinations which were mood congruent. Cognitive functions were intact, with good intelligence and fair insight. She acknowledged her bloodletting and drinking behaviors as inappropriate and expressed a desire to overcome her urge to consume blood.

Diagnosis of Ms. A was challenging as her features did not fit existing diagnostic categories though there were symptoms of NSSI, clinical vampirism, psychotic spectrum, and impulse control disorders. She was provisionally diagnosed as a possible case of *hemomania* as her behaviors were similar to the proposed criteria given by Kandeger et al. [5]. However, collecting and consuming blood from others is a unique feature that does not conform to the proposed criteria of *hemomania*. She also fulfilled DSM‐5 criteria [3] for major depressive disorder (recurrent, with psychotic features), BPD (marked by impulsivity and emotional dysregulation), and she had few schizotypal traits (magical thinking and perceptual abnormalities) but did not meet the total features of schizotypal personality disorder.

She was treated with sertraline (up to 150 mg), propranolol (20 mg/day), olanzapine (10 mg/day), and sodium valproate (300 mg/day), which was later replaced by lithium (400 mg/day), due to suicidal ideation and pregnancy plans. She also received iron and zinc supplements, cognitive–behavioral therapy (CBT), relaxation training, couple counseling, and family therapy. Although structured dialectical behavior therapy (DBT) was not feasible due to her refusal to participate in group sessions, targeted emotion‐regulation and distress‐tolerance strategies contributed to reducing her emotional reactivity and episodes of bloodletting. With regular monitoring, *hemomanic* behaviors and depressive symptoms decreased, though her marital stress persisted. Lithium was later discontinued due to elevated serum levels (1.2 mmol/L).

She was discharged after 40 days of hospital stay. On 1 month follow‐up, mood and bloodletting behavior improved significantly, and she was very eager to get pregnant. But her pregnancy test came negative, which increased her depressive and hemomanic symptoms again. She did not continue regular follow‐up, and her compliance to medication and adherence to therapy sessions was poor.

## 3. Discussion

In our reported case, Ms. A demonstrated a recurrent inability to control urges to view, withdraw, collect, and consume blood from herself and others. There was buildup of inner tension, discomfort, or arousal immediately before the act of bloodletting and blood drinking and experience of gratification, emotional relief, and pleasure immediately after the act. This behavior pattern aligns with the proposed term of “*hemomania*” by Kandeger et al. [2], where he reported two cases with similar behavior and suggested that *hemomania* can be conceptualized as an impulse control disorder. While *hemomania* is not recognized in the DSM‐5, her behaviors have distinctive features that needs separate clinical attention, with differentials including NSSI, clinical vampirism, psychotic spectrum, and impulse control disorders.

Glenn and Klonsky [1] highlighted that NSSI often includes a desire to see blood as a form of emotional regulation, and a recent systematic review conceptualized NSSI as a behavioral addiction as there is often compulsive urges, loss of control, persistence, and concern over the behavior [7]. A meta‐synthesis identified that self‐harm may function not only to obtain relief but also to connect with others when there is a need to express affect and the inability to communicate it verbally, which is relevant in this case [8]. However, the severity, persistence, and ritualistic nature of behaviors in this case go beyond typical NSSI presentations. In a study conducted among 130 individuals with NSSI, 30% had hemomanic behaviors, among them 6.2% and 2.3% individuals reported bloodletting and drinking blood, respectively [5], which indicates the overlapping symptoms of NSSI and *hemomania* though none of them reported collecting blood from another individual, which is a distinct feature in our case.

Ms. A’s behavior of collecting and consuming blood—sometimes extended to drawing blood from other individuals and animals, which is rare and disturbing. These actions were often associated with pleasure, calmness, and, in some instances, sexual gratification. This presentation bears partial resemblance to Renfield syndrome (clinical vampirism), a rare behavioral condition involving compulsive blood drinking linked with psychosexual satisfaction [9], though this historical/descriptive label derived from literary sources and not a validated psychiatric diagnosis. However, established descriptions of this syndrome consist of persistent and identity‐linked fantasies and delusional beliefs related to blood or transformation. Ms. A had none of these features, and the blood‐consuming behaviors occurred in the context of affective dysregulation and impulsivity. Although elements of sexual gratification were reported, it was neither consistent nor primary, therefore, “Renfield syndrome” was excluded. Previous reports describe two males who forcefully obtained blood from others and were diagnosed with dissociative identity disorder and PTSD [10] and dissociative personality disorder [11]. In contrast, Ms. A collected blood consensually from her boyfriend and sister, although she admitted to killing a cat and consuming its blood once—a behavior she later regretted. Notably, there were no features suggestive of dissociation or antisocial personality disorder.

Although Ms. A experienced psychotic symptoms during severe depressive episodes, they were mood‐congruent, episodic, and her reality testing was intact. The blood‐related behaviors were not driven by passivity phenomenon or commanding hallucination; rather, she described them as internally generated urges which she considered inappropriate and unhealthy and adopted multiple neutralizing behaviors to control them (recording and watching videos and drawing with blood). Additionally, there was no longitudinal evidence of functional impairment; hence, the overall clinical picture does not support a primary psychotic disorder. The blood‐related behaviors were episodic, triggered by stress, followed by subjective relief, suggesting a maladaptive coping mechanism and problems with impulse control [3].

The coexistence of major depressive disorder and BPD complicated her illness course. BPD is strongly associated with NSSI and impulsive self‐damaging behaviors [12], providing a partial explanatory framework for Ms. A’s actions. Consistent with this, about 57.7% participants with NSSI fulfilled criteria for major depressive disorder, and odds of having BPD was much higher among individuals who had *hemomania* (OR: 4.76; 95% CI: 1.557–14.548) compared to those who had NSSI without *hemomanic* behaviors [5]. Her bloodletting and blood drinking behavior increased with the severity of her depression, which can be partially explained by Bloodlust [13] who historically explained human bloodlust as a positive response to the sight, feel, and taste of blood during heightened emotional states. In addition, her magical thinking, perceptual distortions, and family’s shared unusual beliefs suggest a schizotypal trait and an environment that may have normalized or reinforced her behavior, though her personality features did not fulfill the criteria for schizotypal disorder in DSM‐5 [3]. Considering the psychopathology in context of her early childhood adversities, the blood‐related behaviors may represent a somatic response to being overwhelmed or an attempt to reclaim control over her body, though the interpretations are tentative.

The management of *hemomania* poses significant challenges due to the lack of evidence‐based guidelines. In this case, management focused on treating the psychiatric comorbidities through a combination of pharmacotherapy—including SSRIs, antipsychotics, and lithium—alongside psychotherapy such as DBT, CBT, and family therapy, with close clinical monitoring. Similar therapeutic approaches were reported elsewhere such as SSRI and anti‐psychotics [2] and mood stabilizers [11]. Nonpharmacological interventions like relaxation training, psychoeducation, and couple and family therapy were helpful in addressing her maladaptive beliefs and relational conflicts. Though DBT is the treatment of choice for BPD, a reasonable alternative is good psychiatric management (GPM), which offers a pragmatic and evidence‐based generalist approach that is feasible in resource‐constrained contexts [14, 15]. Despite partial symptomatic improvement, relapse following psychosocial stressors emphasizes the chronic and relapsing nature of her condition.

To our knowledge, published reports describing a similar presentation from South Asia are scarce. Despite resource constraints, careful diagnostic evaluation and comprehensive interventions led to initial improvement. Limitations include the absence of standardized psychometric assessments and inconsistent follow‐up. While a single case cannot be generalized, the lack of formal diagnostic criteria and clinical guidelines underscores the need for further research and increased professional awareness of this rare condition.

## 4. Conclusion

Although rare, recurrent bloodletting and blood consumption behaviors described under the proposed term *hemomania* can be potentially life‐threatening and are associated with significant psychiatric comorbidities. This case underscores the need for careful differential diagnosis, empathic engagement, and individualized treatment planning. Broader clinical awareness and future research are essential to better understanding and treating this emerging behavioral syndrome.

**Nomenclature**
NSSI: Nonsuicidal self‐injury
DSM‐5: Diagnostic and Statistical Manual of Mental Disorders, Fifth Edition
BPD: Borderline personality disorder
DBT: Dialectical behavior therapy.

## Author Contributions

**Sifat E. Syed:** conceptualization, writing – original draft, writing – reviewing and editing, visualization, supervision. **Nishat Tamanna Nur and Shabnam Saba:** clinical management, data curation, writing – original draft. **Nahid Mahjabin Morshed:** supervision.

.

## Funding

We did not receive any funding for this work.

## Ethics Statement

The Institutional Review Board of the Bangladesh Medical University does not require approval for case reports. Written informed consent was obtained from the patient for the publication of this case report and accompanying images. The patient reviewed the images selected for publication and consented to their use for scientific purposes. All reasonable efforts have been made to protect the patient’s privacy and confidentiality. Identifying information has been omitted or modified where appropriate. However, because of the unusual nature of the clinical presentation, complete anonymity cannot be guaranteed.

## Conflicts of Interest

The authors declare no conflicts of interest.

## Data Availability

The data that support the findings of this study are available upon request from the corresponding author. The data are not publicly available due to privacy or ethical restrictions.
